# HTLV-1 as a contributing factor towards scabies and its systemic sequelae

**DOI:** 10.7189/jogh.13.03057

**Published:** 2023-11-03

**Authors:** Beatrice Cockbain, Carolina Rosadas, Graham P Taylor

**Affiliations:** 1 Department of Infectious Disease, Imperial College London, London, England, UK; 2 Chelsea and Westminster NHS Foundation Trust, London, England, UK; 3 National Centre for Human Retrovirology, Imperial College Healthcare NHS Trust, London, England, UK

**Figure:**
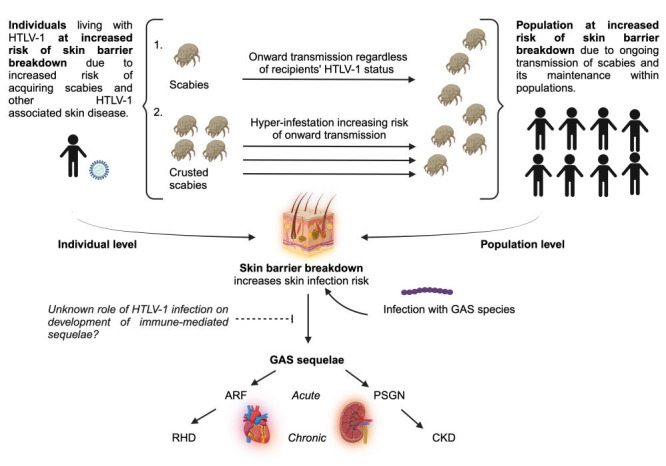
Photo: The hypothesised role of human T-lymphotropic virus type 1 (HTLV-1) as a contributing factor towards scabies and its systemic sequelae at both an individual and population level. Image created using BioRender.comby Cockbain et al.

Scabies is an infestation of *Sarcoptes scabiei* var *hominis*, a microscopic ectoparasite mite with no non-human reservoir. Scabies is transmitted through skin-to-skin contact, with higher numbers of mites conferring greater infectiousness. Like human T-lymphotropic virus type 1 (HTLV-1), scabies is an ancient infection, clustering in socio-economically marginalised populations in Africa, Oceania and South America, with outbreaks often found in institutional settings and other overcrowded areas [1]. There are an estimated 565 million annual incident scabies cases and a global point prevalence of 167 million [2]. The global burden of scabies in relation to the discomfort, disfigurement and skin infection (excluding any sequelae of bacterial superinfection) is an estimated 4.8 million disability-adjusted-life-years [2]. Here we present the case for the interplay between HTLV-1 and scabies, and their direct and indirect links to cardiac and renal disease.

Classical, or typical, scabies infection causes pruritus and characteristic lesions. The global prevalence of classical scabies peaks between the ages of five and 25 years [3], attributed to overcrowding and close contact between children, with a further smaller peak in the 70s [3]. Scabies in older age groups often presents atypically, complicating diagnosis and control of outbreaks [3]. Immunosuppression may lead to hyper-infestation and atypical presentations, including hyperkeratotic plaque-like lesions often without pruritus (crusted, “Norwegian”, scabies). This form of scabies is believed to be particularly transmissible due to the high mite burden [3].

HTLV-1, the first human retrovirus discovered, has a global distribution that reflects both ancient and modern human migration [1]. Transmission of HTLV-1 occurs sexually, via infected blood/organ products, needle-sharing, and vertically (predominantly via human milk) [1]. Originally discovered in the context of a haematological malignancy with poor prognosis (adult T-cell leukaemia/lymphoma (ATLL)) and subsequently found to be the causative agent of a disabling progressive neurological condition (HTLV-1-associated myelopathy (HAM)), HTLV-1 is increasingly implicated in other inflammatory conditions including infective dermatitis, uveitis, bronchiectasis, and co-infections, including *Mycobacterium tuberculosis* and *Strongyloides stercoralis* [4,5]. Estimates from 2012 suggested at least 5-10 million people were living with HTLV-1, however the true prevalence is likely far higher as there are no, or only unreliable, data on the majority of the global population [1].

The co-existence of HTLV-1 and scabies is documented in numerous countries (**Figure 1**). The presence of HTLV-1 infection is associated with an increase in all forms of scabies infection, with the strongest association seen in severe, recurrent, or crusted scabies (**Figure 1**). Indeed, some studies have found 70-100% of patients with crusted scabies to be living with HTLV-1 infection (**Figure 1**), with crusted scabies also seen to be an indicator of other HTLV-1-associated disease. Additionally, areas reporting high prevalence of scabies may have a high prevalence of HTLV-1, particularly First Nations communities in central Australia. Within this setting, the frequency of scabies in adult hospital inpatients doubles in those living with HTLV-1 (72/502 (14.2%) vs. 80/994 (8.5%); *P* = 0.001) (**Figure 1**).

**Figure 1:**
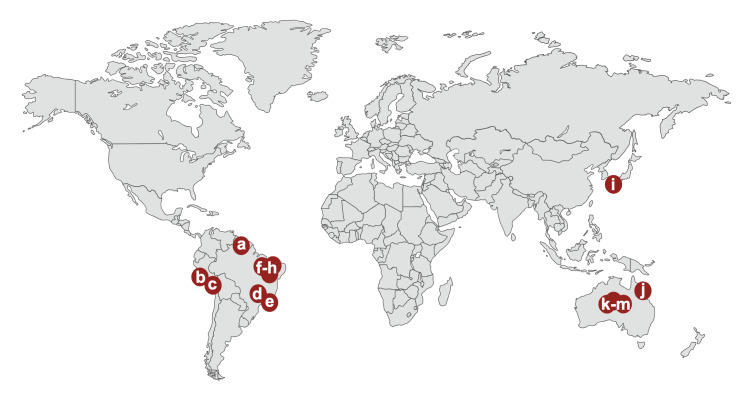
Map of studies demonstrating associations between HTLV-1 and scabies. a) French Guiana: case review of crusted scabies, 6/6 (100%) crusted scabies cases seropositive for HTLV-1 [6]. b) Lima, Peru: prevalence study, 16/23 (69.6%) crusted scabies cases seropositive for HTLV-1; 3/3 (100%) recurrent crusted scabies cases seropositive for HTLV-1 [7]. c) Peru: national HTLV-1 prevalence study, 2/9 (22.2%) with recurrent scabies and HTLV-1 [8]. d) Southeastern Brazil: family study, 2/15 (13.3%) with scabies in HTLV-1 positive group; 0/15 (0%) with scabies in HTLV-1 negative group [9]. e) Rio de Janeiro, Brazil: case-control study, 3/60 (5%) with scabies in HTLV-1 seropositive group (all with HAM/TSP); 0/38 (0%) with scabies in HTLV-1 seronegative group [10]. f) Bahia, Brazil: child prevalence study (n = 30), 70% with HTLV-1 had scabies; 3.3% with HTLV-1 had crusted scabies [11]. g) Bahia, Brazil: scabies case review, 47/91 (51.6%) of all scabies cases HTLV-1 seropositive; 21/23 (91.3%) with crusted scabies cases HTLV-1 seropositive; 8/35 (22.9%) with severe scabies HTLV-1 seropositive; 0/33 (0%) with typical scabies HTLV-1 seropositive [12]. h) Bahia, Brazil: case-control study, 4/179 (2.2%) with scabies in HTLV-1 seropositive group; 0/193 (0%) with scabies in HTLV-1 seronegative group (*P* = 0.053) [13]. i) Kumamoto, Japan: case series. 2/2 (100%) cases of HTLV-1 (both with ATL) and crusted scabies [14]. j) Far North Queensland, Australia: prevalence study, 1/13 (7.7%) HTLV-1 seropositive results when scabies noted on serological tests requests [15]. k) Alice Springs, Australia: Prevalence study, 5/5 (100%) crusted scabies cases seropositive for HTLV-1; 3/9 (33.3%) mild scabies cases seropositive for HTLV-1 [16]. l) Alice Springs, Australia: case-control study, 75/502 (14.2%) with scabies in HTLV-1 seropositive group; 80/994 (8.5%) with scabies in HTLV-1 seronegative group (*P* = 0.001) [17]. m) Alice Springs, Australia: Inpatient case series. 2/2 (100%) scabies admissions HTLV-1 seropositive [18]. Figure created with BioRender.com. HTLV-1 – human T-lymphotropic virus type 1, HAM/TSP – HTLV-1-associated myelopathy/tropical spastic paraparesis, ATL* –* adult T-cell leukaemia/lymphoma.

## CONSEQUENCES OF SCABIES AND HTLV-1 RELATED SKIN BARRIER BREAKDOWN

Scabies lesions, excoriations and HTLV-1 related skin diseases, including infective dermatitis and ichthyosis, may lead to skin barrier breakdown, introduction of bacterial infection and subsequent pyoderma and impetigo [4,19]. Indeed, a 12-fold increased risk of impetigo is seen in children living with scabies in Australia [19]. Bacterial superinfection by *Staphylococcus aureus* and *Streptococcus pyogenes* is common, species whose growth appears to be promoted by scabies mites in animal, in vitro and metagenomic studies.

Impetigo and pyoderma, particularly due to *Streptococcus pyogenes*, are associated with the development of immune-mediated sequelae, including acute rheumatic fever (ARF) and post-streptococcal glomerulonephritis (PSGN), with these implicated in the development of rheumatic heart disease (RHD) and chronic kidney disease (CKD) [20]. In some areas, all PSGN cases were associated with skin, not pharyngeal, infection [20], while familial-clustering suggested common genetic, or infectious factors. Community scabies treatment programmes reduce not only scabies cases but also *Streptococcal* skin contamination and haematuria, an indicator of kidney disease.

The global distribution of ARF, RHD and PSGN mirror those of scabies and HTLV-1, with the highest rates seen in some Australian First Nations populations [1,2,19,21]. The role of HTLV-1 in immune-mediated renal disease is seen in Japan, where 9.9% of patients with primary glomerulonephritis were living with HTLV-1 (compared to 6.6% of local blood donors), and 9/10 of those living with HTLV-1 who were biopsied demonstrated immune complex deposition nephritis [22]. Whether HTLV-1 affects the likelihood of developing PSGN or ARF remains unknown. In a community survey of First Nations’ adults in Central Australia, 33.5% (69/197) of those living with HTLV-1 had chronic kidney disease, compared to 22% (69/313) of those without HTLV-1 (*P* = 0.001) [21]. Although PSGN is not commonly found in this adult population, childhood PSGN can adversely affect the kidney, predisposing adults to chronic renal disease [20], perhaps also adversely affecting renally-mediated blood pressure control. A study of almost 400 adult blood donors in Brazil found those living with HTLV-1 more likely to have hypertension (36.9% (66/179) compared to those without (3.1% (6/193); *P* < 0.001) [4], The exact aetiology of this phenomenon is unknown but it may reflect a role for chronic HTLV-1 mediated inflammation on the cardiovascular system, similar to that seen in other chronic retroviral infections.

## HOW HTLV-1 INFECTION MIGHT DRIVE SCABIES AT INDIVIDUAL AND POPULATIONAL LEVELS

Cutaneous manifestations of HTLV-1 are common, and include cutaneous lymphomas, inflammatory and infective conditions, such as infective dermatitis of HTLV-1 [4]. There is a well-documented association between crusted scabies and HTLV-1, particularly in the context of profound immunosuppression (**Figure 1**). Even in otherwise asymptomatic individuals with HTLV-1, a degree of immune dysregulation is observed [23], which may influence susceptibility to scabies, including more severe infestations. Although the impact of HTLV-1 on mite burden in classical scabies remains unknown, the presence of HTLV-1 co-infection is associated with a higher burden of disease in other infectious conditions, including *Mycobacterium tuberculosis* and *Strongyloides stercoralis* [5].

Individuals with scabies, particularly those with crusted scabies, can transmit to others in close proximity, regardless of the recipients’ immune status. With its atypical presentation, delayed diagnosis and treatment of crusted scabies is common, facilitating ongoing transmission within communities, particularly in the context of poverty and overcrowding. Indeed, the conditions in which scabies infestations thrive: those of poverty, marginalisation and overcrowding, are common in the communities most affected by HTLV-1. More work is required to assess the extent to which scabies and HTLV-1 overlap due to shared social determinants of health compared to underlying biological pathways.

In areas endemic for scabies, such as Fiji and the Solomon Islands, community-wide treatment programmes are often used, as relying only on the treatment of symptomatic individuals may not identify all cases, allowing for ongoing reinfection. Individuals living with HTLV-1 are at heightened risk of both atypical and recurrent scabies infections. Rates of HTLV-1 increase with age, due to the combination of vertical and horizontal transmission [1]. The proportion of children living with HTLV-1 is therefore lower in most communities, while scabies rates among children are generally higher than those seen in adults [3]. However, if adults living with HTLV-1 are more susceptible to recurrent and crusted scabies, and perhaps also to a higher mite burden in classical scabies, transmission of scabies is more likely, including to cohabiting children. This ongoing peer-led transmission, combined with difficulties identifying and treating those at most risk of a high mite burden, facilitates the maintenance of scabies within communities, as can be seen in the illustration above. Rising rates of scabies and emergence of permethrin-resistant cases highlight the need to better understand factors driving scabies outbreaks. This not only includes consideration of social determinants of health such as poverty and overcrowding, but also individual susceptibility to scabies infection, for example concurrent infection with HTLV-1.

## CONCLUSION

High rates of renal dysfunction and rheumatic fever are known to occur not only in scabies-endemic areas but other areas of HTLV-1 endemicity. Skin barrier breakdown is common in HTLV-1, through related skin conditions like infective dermatitis, structural changes like acquired ichthyosis/xerosis, or infestations like scabies. These may lead to the development of bacterial superinfection with species associated with the immune sequelae of acute rheumatic fever and post-streptococcal glomerulonephritis, and the subsequent development of chronic cardiac and renal disease. Antimicrobials for scabies and bacterial skin infections may reduce risks of systemic sequelae but the likelihood of reinfection is high with concurrent HTLV-1.

There are known associations between HTLV-1 and scabies, between scabies and Group A *Streptococcal* skin infections, and between these skin infections and immune-mediated renal and cardiac sequelae. This Viewpoint draws on this evidence and hypothesises additional mechanisms by which HTLV-1 infection could be a driver in the maintenance of scabies and its disease sequelae within communities. Further studies are needed to assess the impact of HTLV-1 not only on scabies, but also on its associated renal and cardiac sequelae, at both individual and population levels.

Although HTLV-1 is not yet curable, its identification allows strategies to be implemented to prevent onward transmission, improving health outcomes for future generations in some of the world’s most marginalised populations.
